# Dextran sodium sulfate (DSS) induces necrotizing enterocolitis-like lesions in neonatal mice

**DOI:** 10.1371/journal.pone.0182732

**Published:** 2017-08-17

**Authors:** Marco Ginzel, Xiaoyan Feng, Joachim F. Kuebler, Christian Klemann, Yi Yu, Reinhard von Wasielewski, Joon-Keun Park, Mathias W. Hornef, Gertrud Vieten, Benno M. Ure, Torsten Kaussen, Jan Hendrik Gosemann, Steffi Mayer, Anne Suttkus, Martin Lacher

**Affiliations:** 1 Center of Pediatric Surgery, Hannover Medical School, Hannover, Germany; 2 Department of Pediatric Surgery, University of Leipzig, Leipzig, Germany; 3 Institute of Pathology, KRH Hannover Northern City Hospital, Hannover, Germany; 4 Department of Nephrology, Hannover Medical School, Hannover, Germany; 5 Institute for Medical Microbiology, RWTH-Aachen, Aachen, Germany; 6 Department of Pediatric Cardiology and Pediatric Intensive Care, Hannover, Germany; Children's Hospital of Los Angeles, UNITED STATES

## Abstract

**Background:**

Necrotizing enterocolitis (NEC) is an inflammatory bowel disease of preterm human newborns with yet unresolved etiology. An established neonatal murine model for NEC employs oral administration of lipopolysaccharides (LPS) combined with hypoxia/hypothermia. In adult mice, feeding dextran sodium sulfate (DSS) represents a well-established model for experimental inflammatory bowel disease. Here we investigated the effect of DSS administration on the neonatal murine intestine in comparison with the established NEC model.

**Methods:**

3-day-old C57BL/6J mice were either fed formula containing DSS or LPS. LPS treated animals were additionally stressed by hypoxia/hypothermia twice daily. After 72 h, mice were euthanized, their intestinal tissue harvested and analyzed by histology, qRT-PCR and flow cytometry. For comparison, adult C57BL/6J mice were fed with DSS for 8 days and examined likewise. Untreated, age matched animals served as controls.

**Results:**

Adult mice treated with DSS exhibited colonic inflammation with significantly increased Cxcl2 mRNA expression. In contrast, tissue inflammation in neonatal mice treated with DSS or LPS plus hypoxia/hypothermia was present in colon and small intestine as well. Comparative analysis of neonatal mice revealed a significantly increased lesion size and intestinal Cxcl2 mRNA expression after DSS exposure. Whereas LPS administration mainly induced local neutrophil recruitment, DSS treated animals displayed increased monocytes/macrophages infiltration.

**Conclusions:**

Our study demonstrates the potential of DSS to induce NEC-like lesions accompanied by a significant humoral and cellular immune response in the small and large intestine of neonatal mice. The new model therefore represents a good alternative to LPS plus hypoxia/hypothermia administration requiring no additional physical stress.

## Introduction

Necrotizing enterocolitis (NEC) represents a devastating intestinal inflammation in preterm infants and is associated with high morbidity and mortality. It’s pathogenesis still remains elusive but epidemiologic observations strongly suggest a multifactorial etiology [1]. Several risk factors such as prematurity, formula feeding, the presence of certain bacteria and physical stress have been proposed to contribute to the disease pathogenesis. The current rodent models of NEC aim at mimicking these factors by feeding animals with lipopolysaccharide (LPS)-supplemented formula milk in combination with repeated exposure to hypoxia and/or hypothermia [2, 3]. Feeding and postprandial hypoxia had been shown to synergistically promote intestinal inflammation in neonatal mice [4]. However, the role of hypoxia in the pathogenesis of NEC in human preterm neonates has been questioned since NEC usually develops several weeks after birth and not perinatally, when asphyxia and cold stress might occur [5, 6].

Neonatal NEC resembles intestinal bowel disease in adults with regards to pathophysiological processes including an altered mucosal defense, disruption of commensal bacteria as well as an inappropriate immune response [7]. In adult mice, oral dextran sodium sulfate (DSS) administration serves as model for inflammatory bowel disease [8–10]. Therefore, we studied the potential of DSS-induced mucosal tissue damage to serve as a model for NEC in newborn mice.

## Materials and methods

### Ethics statement

All animal experiments were performed in compliance with the German animal protection law (TierSchG) and were approved by the local animal welfare committee (approval 12/0687 and 12/0927 of the Niedersächsische Landesamt für Verbraucherschutz und Lebensmittelsicherheit Oldenburg, Germany). Mice were bred locally and held under specific pathogen-free or germ-free conditions at the Hannover Medical School animal facility.

### Adult DSS mouse model

8-week-old C57BL/6J mice (weight: 18–22 g) received food and drinking water ad libitum. Colitis was induced by 3% (w/v) DSS (molecular weight 40 kDa; Sigma Aldrich, St. Louis, MO, USA) added to the drinking water. Age-matched animals served as controls. Mice were euthanized after an experimental period of eight days, in cases of rectal bleeding, or >10% weight loss. The complete intestines were evaluated macroscopically for signs of colitis (intestinal segments of bowel necrosis, intestinal hemorrhage and perforation). 1 cm of the proximal jejunum, distal ileum and colon was removed and stored in 100 μL RNAlater (Qiagen, Hilden, Germany) at -80°C for subsequent total RNA isolation and qRT-PCR for Cxcl2. The remaining intestinal tissue was fixed for three days in 10% buffered paraformaldehyde (PFA) and embedded in paraffin, sectioned (4 μm) and stained with hematoxylin-eosin using a standard protocol. Small and large intestinal tissue was investigated histologically for intestinal damage as previously described [11].

### Neonatal mouse model

72 h after birth, litters of 6 to 12 C57BL/6J neonatal mice were separated from the mother and placed on a heating plate (37°C). Animals were randomly divided into two groups (i) LPS: Formula feeding supplemented with LPS from *E*. *coli* 0127:B8 (10μg/g body weight, Sigma Aldrich) and exposure to hypothermia and hypoxia as previously described [11]; (ii) DSS: Formula feeding supplemented with 3% (w/v) DSS without physical stress. Age-matched breast fed animals served as controls. Starting 3 h after separation from the dam, pups of all groups were fed by orogastric gavage every 3 h with 50 μl (~115 kcal/kg/day) of 33% Esbilac formula (PetAg, Hampshire, IL, USA) using a 24G silicon catheter (Vygon, Aachen, Germany). To account for the increase in body weight, feeding-volume was increased daily as tolerated (50, 60 and 70 μl on day 1, 2 and 3, respectively).

During the entire experiment (72 h) animals were monitored every 3 h for clinical signs of NEC such as abdominal distension, apnea, rectal bleeding and lethargy. Body weight was assessed twice daily. Animals were euthanized after 72 h or if clinical signs of NEC occurred. Small and large intestines were removed and evaluated macroscopically for signs of NEC (intestinal segments of bowel necrosis, intestinal hemorrhage and perforation).

In a first set of experiments (DSS n = 10, LPS n = 10, controls n = 9), the entire intestine was harvested and fixed for 3 days in 10% PFA for histologic analysis. One mouse of the LPS group and 6 mice of the DSS group exhibited signs of NEC between 60 and 72 h, thus were euthanized earlier.

Therefore, in a second set of experiments (DSS n = 8, LPS n = 8, controls n = 8), exposure to LPS or DSS was reduced to 60 h to ensure a more homogenous study population. Small intestinal tissues were processed to isolate intestinal epithelial cells (IECs) and lamina propria lymphocytes (LPLs). In addition, 1 cm of the proximal jejunum, distal ileum and colon was removed and stored in 100 μL RNAlater (Qiagen, Hilden, Germany) at -80°C to allow RNA isolation and qRT-PCR.

In a third set of experiments (DSS n = 2, LPS n = 2, controls n = 2,), animals were treated with DSS or LPS for only 36 h to histologically investigate early apoptosis of small and large intestines prior to the occurrence of necrosis.

### Histologic injury score of NEC in neonatal mice

The entire bowel was investigated for location, size and severity of intestinal lesions as well as the presence of intraluminal blood and bacteria using a light microscope (Leica, Wetzlar, Germany, magnification 200x). The longitudinal length of intestinal defects (= *size* of intestinal lesions) was measured using a scale bar. The *severity* of intestinal lesions was assessed by two blinded investigators (RvW, JKP) using an intestinal tissue injury score (0–4) based on the histologic injury scoring system of Caplan et al. [2, 11]: Grade 0: intact villi; 1: superficial epithelial cell sloughing; 2: mid-villous necrosis; 3: complete villous necrosis; 4: transmural necrosis. Samples with histological scores of 2 or higher were considered positive for NEC. Grading of *intraluminal blood and bacteria* was carried out according to the guidelines of the Sydney system [12]. In brief, luminal erythrocytes and bacterial growth in a distance of a maximum of 1.5 cm next to intestinal lesions were scaled as follows: None (grade 0); mild (grade 1): presence of single organisms or erythrocytes; severe (grade 3): presence of a large amount of bacteria or erythrocytes. A moderate grade was a condition between these two (grade 2).

### qRT-PCR of the pro-inflammatory chemokine Cxcl2

Total RNA was isolated from whole organ jejunal, ileal and colonic samples of adult and neonatal mice using the RNeasy mini kit (Qiagen, Hilden, Germany) according to the manufacturer’s instructions as described previously [11]. The concentration and purity was determined with a Nanodrop 2000 spectrophotometer (Thermo scientific, Waltham, MA, USA), integrity was analyzed on a 2% agarose gel.

In brief, RNA was transcribed to cDNA using the high capacity RNA to cDNA kit (Qiagen). The mRNA expression levels of the chemokine Cxcl2 (macrophage inflammatory protein-2, MIP2) were measured using the appropriate Taqman primer (Mm00436450_m1, life Technologies, Darmstadt, Germany). Hypoxanthine-guanine phosphoribosyltransferase (HPRT, Mm01545399_m1, life Technologies) expression was measured for normalization as it reaches the exponential phase at similar cycles as the target gene. qRT-PCR was performed using the Applied Biosystems PCR System “Step one Plus” (life Technologies).

### Isolation of intestinal epithelial cells (IECs) and lamina propria lymphocytes (LPLs)

For the in vitro toxicity test, IECs were isolated from 4 days old untreated neonatal mice. For the analysis of myeloid cells in the *lamina propria* of neonatal mice after NEC induction (treated animals) by flow cytometry, small intestines were obtained as described above. Small intestinal samples were processed separately per animal, opened longitudinally and washed 3 times in 20 mL 4°C PBS^-/-^ [13]. They were cut into 1–2 mm long segments and incubated in 25 mL of a 5 mM EDTA PBS^-/-^ solution (Applichem, Darmstadt, Germany) for 20 min at 37°C and 200 rpm on an orbital shaker. After 10 min of sedimentation at 4°C, supernatants and sediment were separated. Sediments, containing untreated *IECs*, were resuspended in 10 mL 4°C smooth muscle growth medium-2 (SmBM-2) (supplemented with growth factors according to the manufacturer’s instructions; Lonza, Basel, Switzerland). The cell suspension was poured through a 40 μM cell strainer (BD Bioscience, San Jose, CA, USA) followed by centrifugation for 5 min at 4°C and 300 x g. The pellet was resuspended in SmBM-2 for culturing. IECs of treated animals were discarded.

The remaining intestinal pieces of treated animals were washed in 10 mL RPMI 1640 media (Lonza) containing 10% fetal calf serum (FCS, Lonza), followed by enzymatic digestion in 5 mL RPMI 1640 media containing 10% FCS, 5 mM HEPES (PAA, Pasching, Austria) and 100 μg/mL Liberase (Roche, Basel, Switzerland) for 60 min at 37°C and 200 rpm on an orbital shaker. The solution was centrifuged for 5 min at RT and 300 x g. The supernatant was discarded and the pellet was resuspended in 4 mL of 40% Percoll (GE Healthcare, Buckinghamshire, GB). This solution was underlayed with 5 mL of 70% Percoll. The interphase, containing the *lamina propria lymphocytes*, was collected and subjected to flow cytometry.

### Flow cytometry

For flow cytometry measurements, a minimum of 1 x 10^5^ living cells was evaluated according to standard protocols [14]. In brief, fixable Viability Dye (eBioscience, San Diego, CA, USA) was used to exclude dead cells and the following antibodies (all purchased from eBioscience) were employed for staining in the presence of anti-FcRII/III mAb (clone 2.4G2; Biolegend, San Diego, CA, USA): CD45_30F-11, CD11b_M1/70, CD11c_N418, e-Cad_36/E-Cadherin, F4/80_BM8, Ly6-C_HK1.4, Ly6-G_1A8, I-A/I-E_M5/114.15.2. Neutrophils were identified by a gating strategy according to Rose et al (CD45^+^, CD11b^+^, SSC^int/hi^, Ly6-C^+^ and Ly6-G^+^) [15] and intestinal monocytes/macrophages populations and nomenclature according to Bain et al. [16]. Samples were acquired on a FACS CANTO II flow cytometer (BD, Franklin Lakes, NJ, USA) and analyzed with KALUZA software (Beckman Coulter, Krefeld, Germany). Total cell numbers were calculated based on gated and total isolated cells.

### Culturing and stimulation of intestinal epithelial cells for viability measurements

Wells of a 96 well plate were covered with matrigel (BD Bioscience, Franklin Lakes, NJ, USA) according to the manufacturer’s instructions. The purity of isolated cells was analyzed by flow cytometry. 100.000 cells with a purity of more than 90% were seed in each well in 250 μL SmBM (Lonza) medium. After 24 h, cells were washed once with 4°C PBS^-/-^. Afterwards, cells were incubated for 72 h in SmBM or SmBM supplemented with 3% DSS (w/v). 1% Triton-X treated cells served as dead cell controls. Viability was measured using the ApoLive-Glo^™^ kit (Promega, Mannheim, Germany).

### Immunohistochemistry for caspase 3

Histological slides of small intestines were stained with a monoclonal rabbit anti-mouse- antibody against caspase 3 (Cell Signaling Technology, Danvers, Ma, USA) using a standard protocol. In brief, sections were deparaffinized for 10 min with xylene and rehydrated with ethanol and aqua dest. After that, sections were placed in 0.01 M citrate puffer and heated in the microwave for 10 min. Samples were blocked with 5% goat serum in TBS for 20 min. The primary antibody was incubated over night at 4°C, followed by three times washing with TBS and incubation with the secondary antibody for 30 min at RT. After 3 more washing steps in TBS, samples were covered in mounting medium with DAPI (Dianova, Hamburg, Germany). The amount of caspase 3 positive cells was determined using a semi-quantitative sore: Samples were evaluated at a magnification of 200x and cells were estimated per field of view (FOV). Grade 0: No caspase 3 positive intestinal cells (0 cells / FOV); grade 1: presence of single or isolated positive cells (1–5 cells / FOV); grade 2: moderate amount / groups of positive cells (6–50 cells / FOV); grade 3: massive positive area of cells (> 50 cells / FOV).

### Statistical analysis

Results are expressed as means ± standard deviation (SD). For body weight changes, qRT-PCR, flow cytometry and *in vitro* viability, one way analysis of variance (ANOVA) with Bonferroni post hoc tests was used. Diameter of intestinal lesions was assessed by student t-test, NEC and cleaved caspase 3 scores by Kruskal-Wallis one way analysis of variance on ranks. The incidence of NEC was calculated using Chi Square test. For comparison of luminal blood and bacteria scores, Mann-Whitney-U test was performed. Graphs were designed with GraphPad Prism (La Jolla, CA, USA), p-values were calculated with the software SPSS (Version 24, IBM^®^, Armonk, NY, USA) and considered significant when < 0.05.

## Results

### Following DSS administration to adult mice inflammation is confined to the colon

**Fig 1:**
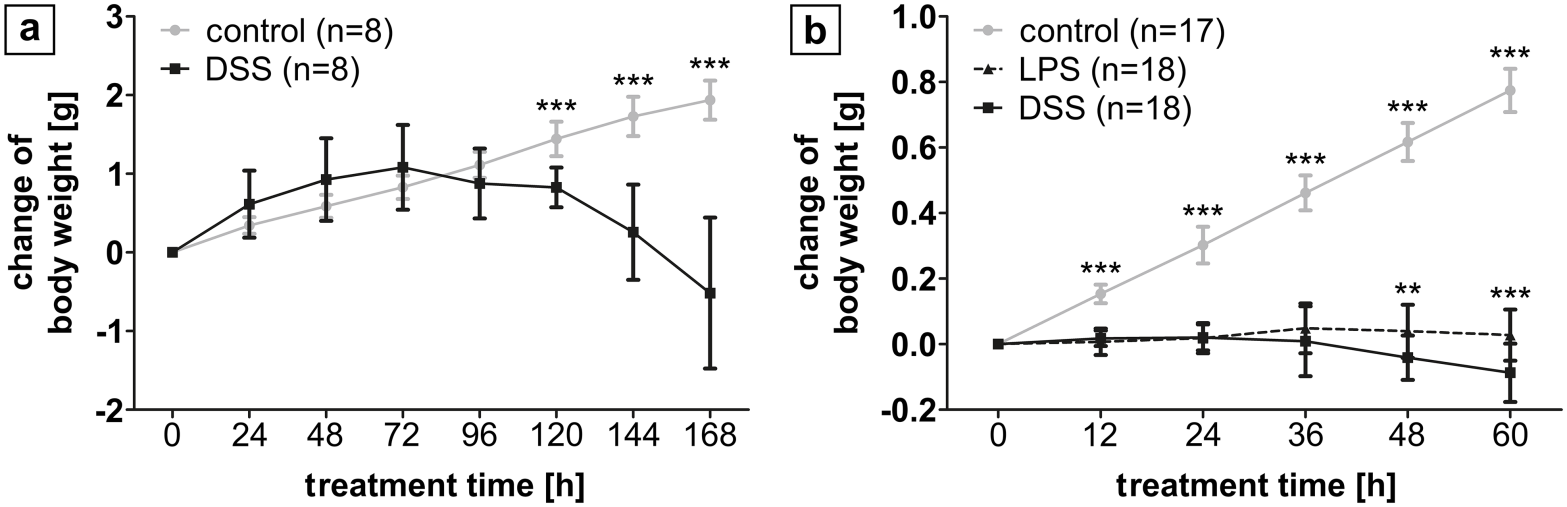
Change in body weight following treatment of adult and newborn mice. (a) In adult mice, controls continuously gained weight over 8 d, whilst after DSS treatment initial weight gain reversed into weight loss, significantly different from controls from the 5^th^ experimental day on. (b) In neonatal mice, controls also continuously gained weight over 60 h. Animals from the LPS and DSS group gained weight in a similar matter for 36 h followed by a significant weight loss, which was more pronounced for DSS (p < 0.001). ** p < 0.01, *** p < 0.001.

**Fig 2:**
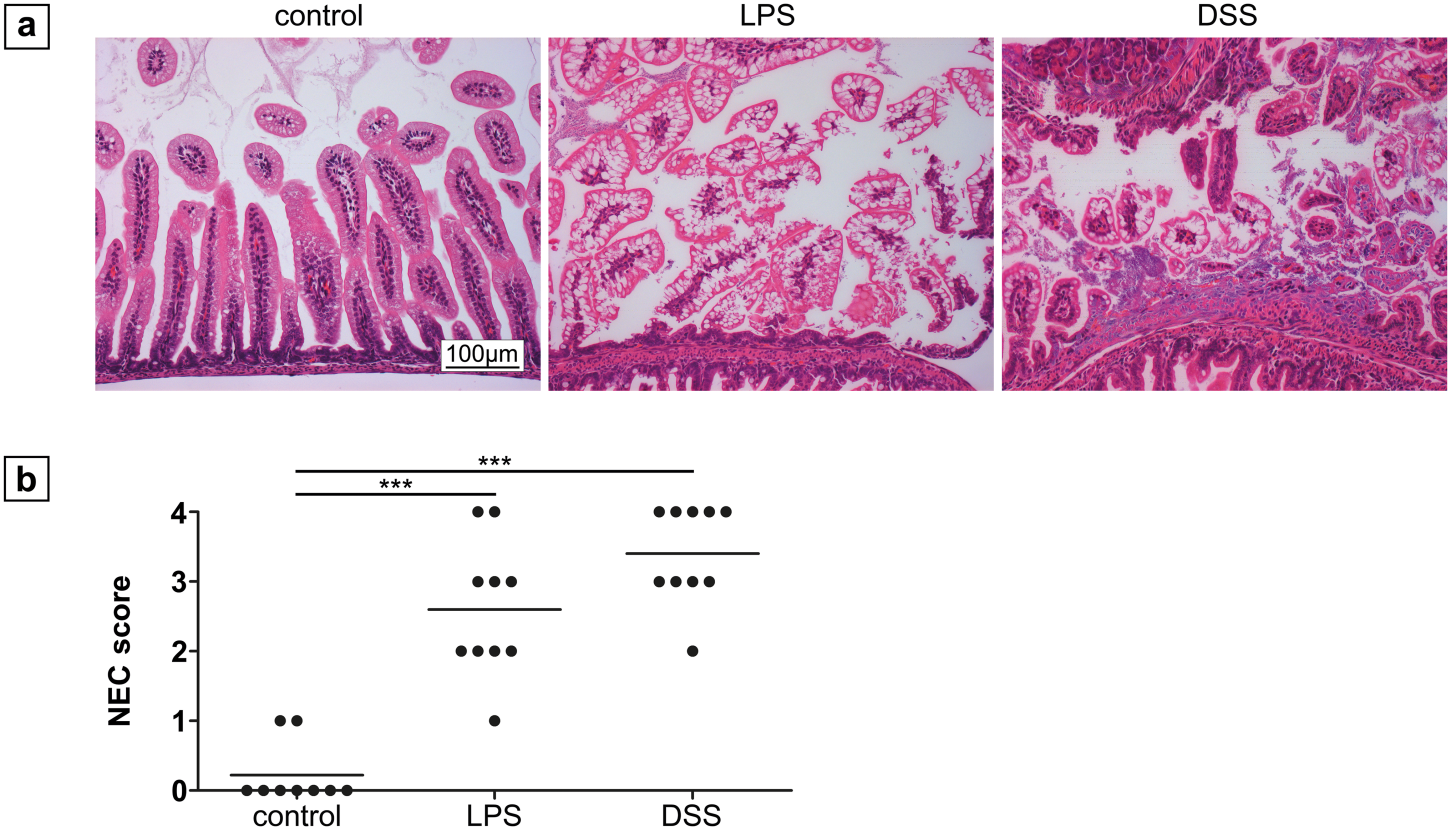
Normalized mRNA expression of Cxcl2 in total intestinal tissue. (a) In adult mice (n = 6) treated with DSS, Cxcl2 expression significantly increased in colon samples only. (b) In neonatal mice (n = 6), Cxcl2 expression was significantly higher in the DSS group as compared to LPS (jejunum, ileum) and controls (jejunum, ileum, colon), respectively. LPS and controls did not differ. * p < 0.05, ** p < 0.01, *** p < 0.001.

After seven days of DSS administration to adult mice, three of eight mice (37.5%) developed visible rectal bleeding as a clinical sign of intestinal inflammation and were euthanatized prior to the end of the experiment. The body weight of control animals increased continuously from 18.87 ± 0.7 g to 20.81 ± 0.8 g after eight days. In contrast, animals exposed to DSS only initially gained weight (initial weight 20.6 ± 2.6 g; weight increase during the first 72 h 0.95 ± 0.5 g) before they significantly lost weight until the end of the experiment (-1.0 ± 0.9 g; Fig 1A). Macroscopic signs of inflammation were exclusively detected in the colonic tissue. Furthermore, colonic Cxcl2 expression exhibited a 46-fold increase after DSS exposure compared to controls (p = 0.02), whilst Cxcl2 expression remained unaltered in jejunal and ileal samples (Fig 2A).

#### DSS treatment of neonatal mice results in more severe weight loss than LPS treatment

No spontaneous mortality was observed in newborn mice exposed to DSS or LPS. However, one animal of the LPS group (10%) and six animals of the DSS group (60%) had to be euthanized prior to the endpoint of the experiment (at 60 and 72 h treatment). In the second (60 h duration) and third (36 h duration) set of experiments, no animal had to be euthanized prior to the experimental endpoint.

After separation from the dam, the mean weight of neonatal mice was 1.98 ± 0.55 g. Control animals continuously gained weight throughout the entire experiment (0.9 ± 0.03 g). During the first 36 h after DSS/LPS treatment, animals gained weight in a similar manner (DSS 0.01 ± 0.1 g, p = 0.47; LPS 0.05 ± 0.1 g, p = 0.47). Thereafter, animals in both treatment groups significantly lost weight. After 60 h of treatment, mice of the DSS group lost significantly more weight (0.14 ± 0.06 g) than those of the LPS group (0.04 ± 0.06 g; p<0.001; Fig 1B).

### Macroscopical and histological changes

Macroscopical evaluation of the large and small intestinal tissue of animals of the LPS group did not reveal any signs of transmural inflammation (necrosis), but two animals (20%) showed slight reddening of the small intestinal mucosal surface (Panel A in S1 Fig). In the DSS group, intraluminal blood was observed in seven (70%) and tissue necrosis in two (20%) animals (Panel B and C in S1 Fig).

**Fig 3:**
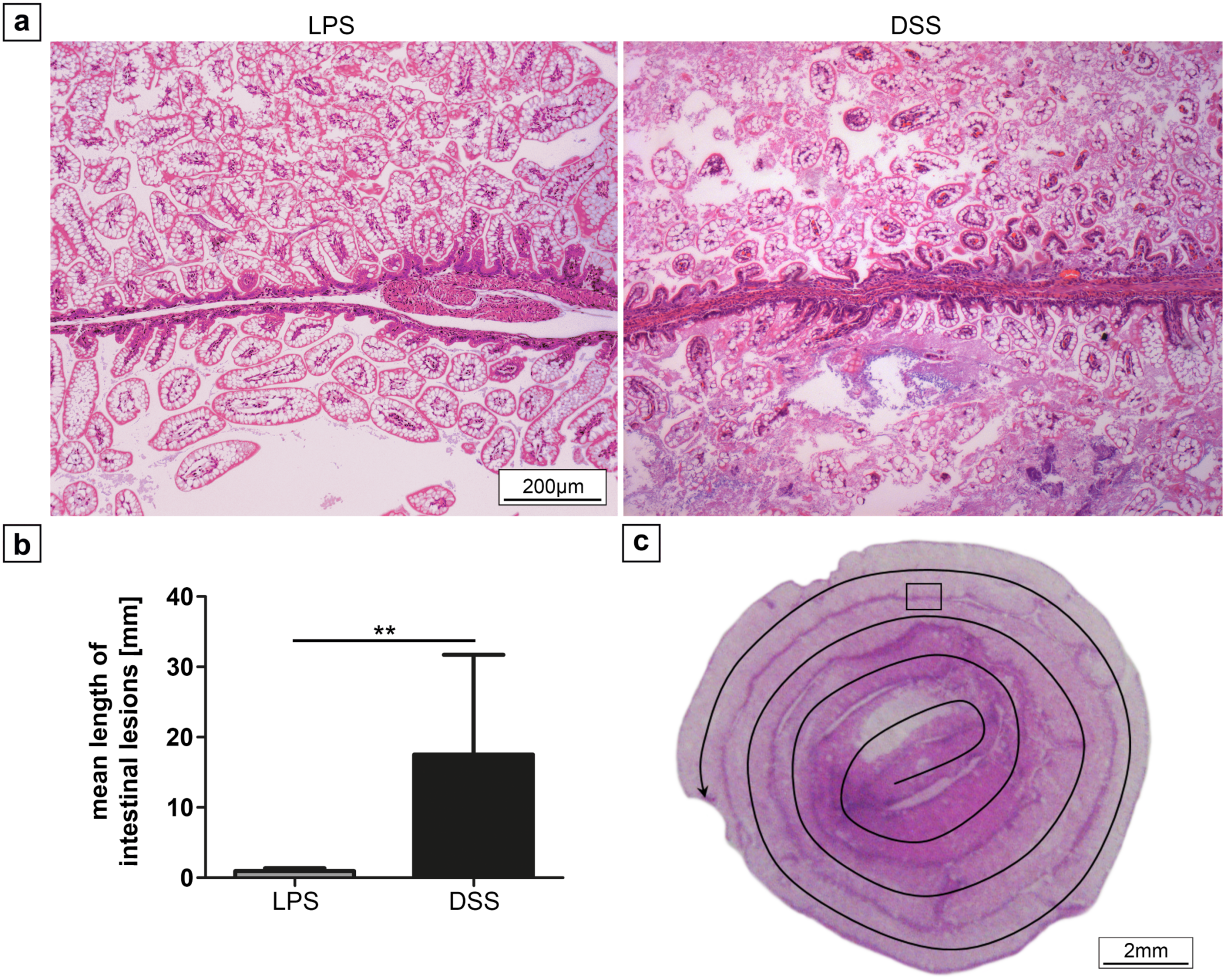
Histological changes of the small intestinal tissue after 72 h treatment. (a) Representative ileal sections of neonatal mice from controls, the LPS, and DSS group (magnification 200x), showing healthy villi-structure versus injured intestinal segments (LPS: NEC score 2; DSS: NEC score 4) in the treatment groups. Severity of intestinal lesions and the incidence of NEC (b) as assessed by the NEC score was similar in both groups (n = 10). *** p < 0.001.

**Fig 4:**
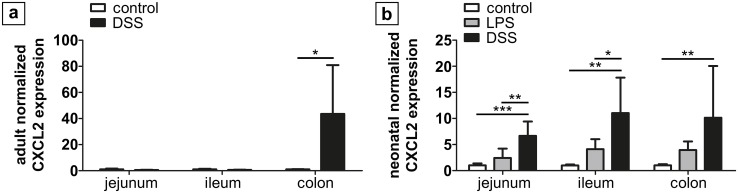
Extension of intestinal lesions in neonatal mice after LPS and DSS treatment. (a) Representative histological sections of intestinal lesions (magnification 100x) after LPS and DSS treatment. Extensive lesions were only detected in DSS treated animals, affecting up to three adjacent intestinal loops of the formed snail. Evaluation (b) revealed significantly larger lesions after application of DSS compared to LPS treatment. (c) Histological sample illustrating the method of length measurement of intestinal lesions. In the central region, the jejunum is located (beginning arrow), the outer loops represent the ileum. The rectangle illustrates the area shown in (a), the arrow indicates the course of length measurement. ** p < 0.01.

Histological analysis of the small intestinal tissue revealed lesions in both treatment groups with a similar severity and incidence as assessed by the Caplan score (NEC ≥ 2) (DSS, 3.4 ± 0.7 *vs* LPS, 2.6 ± 1.0; p > 0.05; Fig 3A and 3B). However, the lesion size was greater in the DSS than in the LPS group (17.5 ± 14.2 mm *vs* 0.9 ± 0.38 mm; p = 0.005; Fig 4A and 4B). Furthermore, the abundance of luminal erythrocytes was higher in the DSS group compared to the LPS group (2.3 ± 0.8 *vs* 0.5 ± 0.7; p < 0.001; S2 Fig). Finally, bacterial growth was more pronounced in the DSS group compared to the LPS group (2.5 ± 0.5 *vs* 1.1 ± 0.6; p < 0.001) with a high correlation between the number of erythrocytes and bacterial growth in the DSS group (Pearson correlation coefficient = 0.76; S3 Fig).

### Increased expression of pro-inflammatory chemokines in the neonatal DSS NEC model as compared to the LPS model

Normalized expression of Cxcl2 was significantly higher in jejunal (6.7-fold; p < 0.001), ileal (11.3-fold; p = 0.002) and colonic (10.1-fold; p < 0.05) tissue in the DSS group in comparison to healthy controls (Fig 2B). In contrast, Cxcl2 expression did not differ between LPS treated animals and controls. Comparing both treatment groups, expression of Cxcl2 was significantly higher after DSS exposure compared to LPS treatment in jejunal (p = 0.005) and ileal (p = 0.03) but not colonic samples (p = 0.3).

### DSS or LPS treatment of neonatal mice leads to differences in the infiltration of pro-inflammatory myeloid cells into the lamina propria

**Fig 5:**
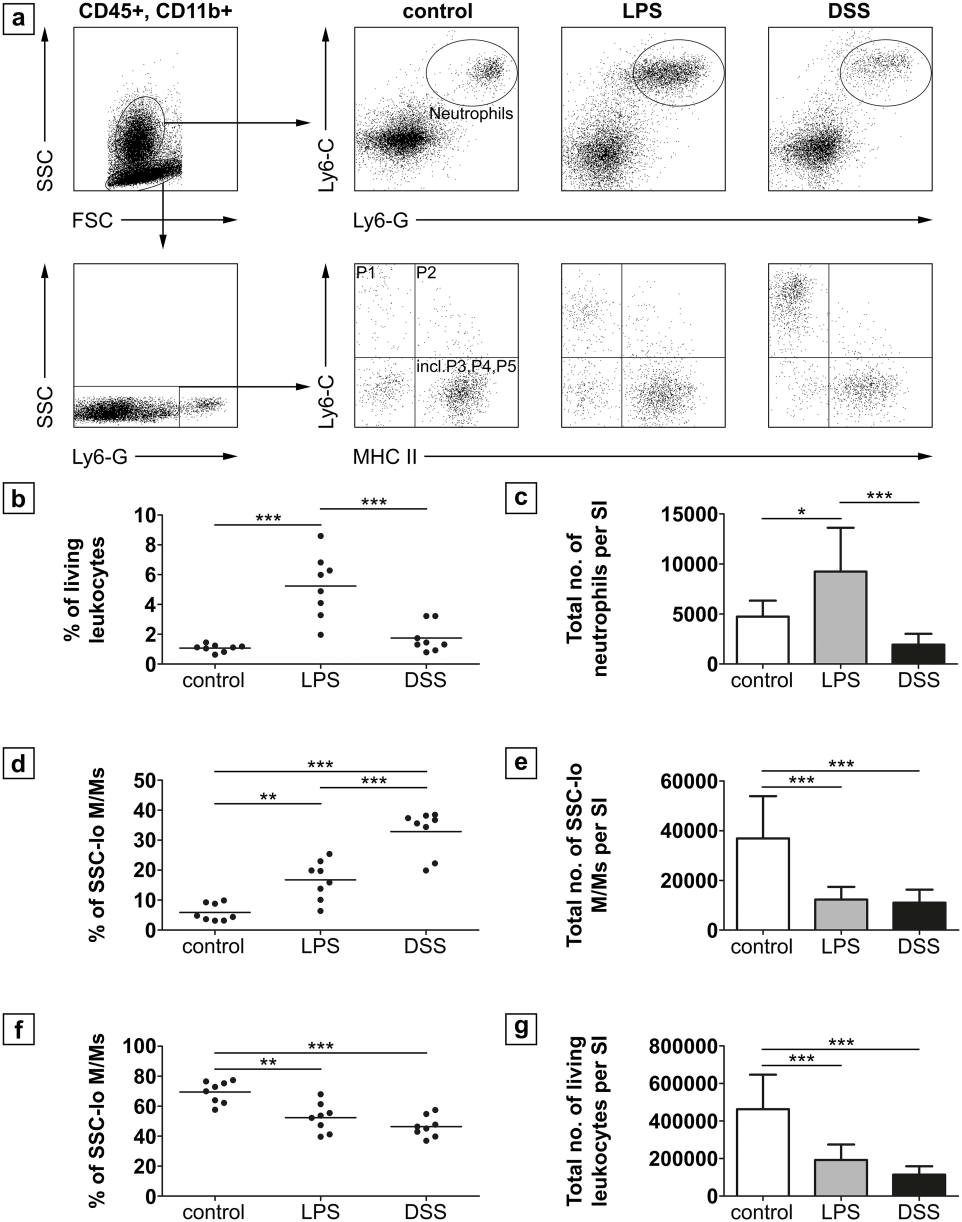
FACS analysis of leukocytes in the lamina propria of the small intestine of neonatal mice. (a) Gated living CD45^+^, CD11b^+^ cells were either further gated on SSC^int/hi^, Ly6-G and Ly6-C double positive cells to identify neutrophils or on SSC^lo^, Ly6-G^-^, Ly6-C and MHC II to identify intestinal monocytes/macrophages (M/M) populations (n = 8 per group). The percentage (b) as well as the total number (c) of neutrophils was significantly higher in the LPS group than in the DSS group and in controls, which did not differ among each other. Conversely, the percentage (d) of Ly6-C^hi^ “inflammatory monocytes” (P1 gate) was higher in the DSS group as compared to the LPS group and controls. The total number of SSC^lo^ monocytes/macrophages was significantly lower in both treatment groups compared to controls. The percentage of resident macrophages found in the “incl. P3, P4, P5” gate (f) was significantly decreased after treatment compared to controls. (g) Total numbers of living leukocytes was significantly decreased in both treatment groups. * p < 0.05 ** p < 0.01, *** p < 0.001; SI = small intestine.

Total leukocyte numbers (CD45^+^) were 4.6 x 10^5^ ± 1.8 x 10^5^ cells per small intestine (jejunum, ileum) of controls, and were significantly decreased in both treatment groups (DSS: 1.1 x 10^5^ ± 4.5 x 10^4^ cells, p < 0.001; LPS:1.9 x 10^5^ ± 8.2 x 10^4^ cells, p = 0.001; Fig 5G). The absolute number as well as percentage of neutrophils within the fraction of viable leukocytes of controls was 4.7 x 10^3^ ± 1.6 x 10^3^ cells (1.07 ± 0.25%) and did not differ from the DSS group (1.9 x 10^3^ ± 1.1 x 10^3^ cells; 1.74 ± 0.95%; p = 0.996) but was increased in the LPS group (9.2 x 10^3^ ± 4.4 x 10^3^ cells; 5.2 ± 2.1%; p < 0.01 each; Fig 5A and 5C).

The percentage of Ly6C^hi^ “inflammatory” (M1) monocytes/macrophages was significantly increased in the DSS group (32.9 ± 7.4%, p < 0.001) and in the LPS group (16.8 ± 6.5%, p < 0.004) as compared to controls (5.9 ± 2.9%; Fig 5A and 5D). DSS exposure led to a significant higher infiltration of M1 monocytes/macrophages than LPS (p < 0.001). In contrast, total cell numbers of SSC^lo^ monocytes/macrophages were significantly lower in the DSS group (1.1 x 10^4^ ± 0.5 x 10^4^ cells, p < 0.001) and LPS group (1.2 x 10^4^ ± 0.5 x 10^4^ cells, p < 0.001) compared to controls (3.7 x 10^4^ ± 1.6 x 10^4^) but did not differ amongst each other (p = 1; Fig 5E). The percentage of resident tissue monocytes/macrophage populations within the P3, P4, and P5 gate (according to Bain et al. 2013 [14]) was decreased in both treatment groups (DSS: 46.4 ± 7%, p < 0.001; LPS 52.4 ± 9.6%, p = 0.001) as compared to healthy controls (69.5 ± 7.4%; Fig 5F).

### DSS has no toxic effect on intestinal epithelial cells (IECs) in vitro

**Fig 6:**
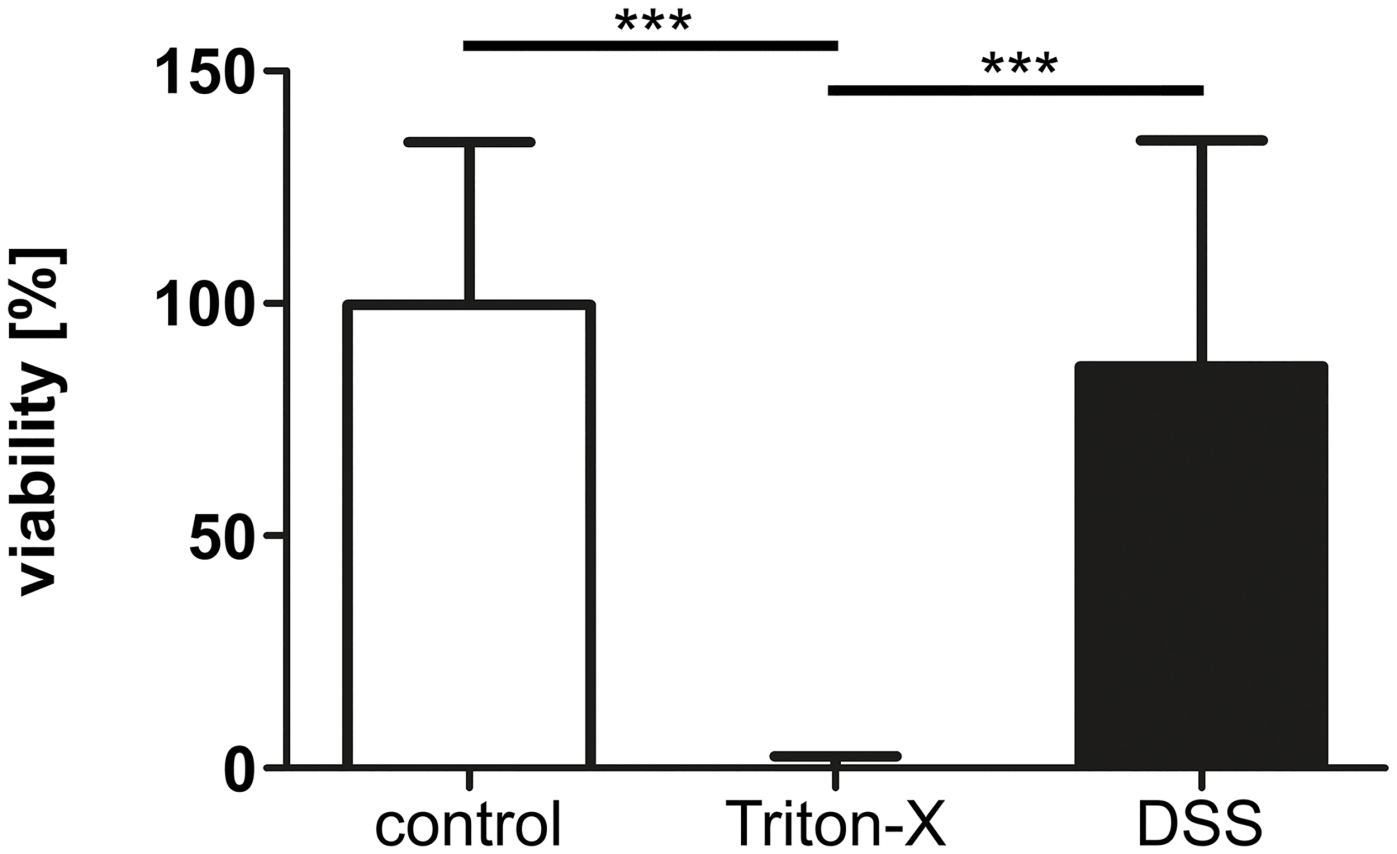
In vitro viability of neonatal intestinal epithelial cells exposed to DSS. Viability of IECs (n = 8) remained unaltered by an exposure to 3% DSS for 72 h in SmBM full medium. 1% Triton-X served as dead cell controls. *** p < 0.001.

No significant difference regarding the viability of intestinal epithelial cells was found after exposure to 3% DSS for 72 h as compared to control cells (Fig 6).

### Enhanced apoptosis in the ileum of neonatal mice after DSS treatment

**Fig 7:**
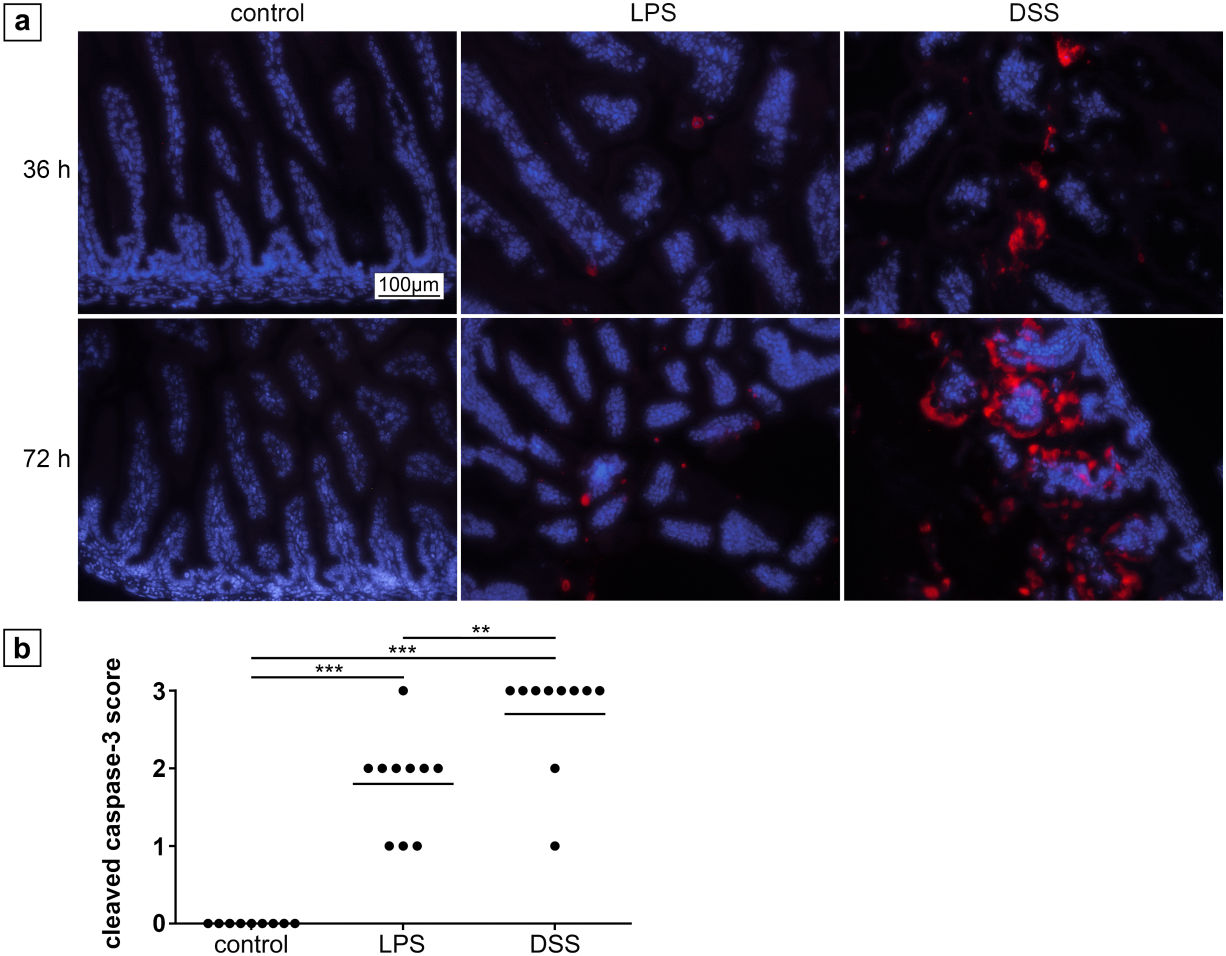
Cleaved caspase-3 staining of the ileum in neonatal mice. (a) Tissue of control animals at the age of 5.5 and 7 days did not show any apoptosis. In contrast, LPS treatment for 36 h led to rare apoptosis, whereas DSS treatment induced moderate apoptosis after the same period of time. After 72 h, increased apoptosis was detected in tissue of LPS treated animals, but to a significantly lesser extent than DSS, which caused a large amount of apoptotic cells. Magnification 200x, cleaved caspase-3: red, nuclei (DAPI): blue. (b) Grading of apoptosis after 72 h. ** p < 0.01, *** p < 0.001.

Healthy controls did not show any signs of intestinal apoptosis by caspase 3 staining (Fig 7A) but different degrees of apoptosis were detected after 36 h and 72 h in the treatment groups, respectively. After 36 h, only a few apoptotic cells (grade 1) were found in the LPS group but moderate apoptosis (grade 2) in the DSS group (Fig 7A). After 72 h, apoptosis significantly increased in both treatment groups as compared to healthy controls (p < 0.001; Fig 7A and 7B). Moreover, apoptosis was significantly higher in the DSS group (grade 3) compared to the LPS group (grade 2; p = 0.006; Fig 7B).

## Discussion

Several animal models have been developed to better understand the underlying pathogenesis of NEC. In a previous study carried out by our group a treatment time of 72 h with LPS combined with exposure to hypoxia/hypothermia was necessary to induce NEC-like mucosal lesions in the intestines of neonatal mice, starting at the age of 4 days [11]. However, the role of hypoxia/hypothermia in the pathogenesis of NEC in human preterm infants has recently been questioned [5, 6]. Thus, we aimed at establishing an alternative NEC model without the requirement for physical stressors. The model presented in this study represents a modification of the DSS model for inflammatory bowel disease in adult mice. To the best of our knowledge this is the first description of the intestinal histological and functional consequences of DSS administration to neonatal mice.

Both, newborn and adult animals exposed to oral DSS significantly lost weight during the second half of the first week of treatment and showed macro- and microscopic signs of intestinal inflammation. Whereas signs of inflammation were exclusively seen in the colon of adult mice, necrotic lesions in neonatal mice were preferentially situated in the small bowel and to a lesser extent in the large intestine. Likewise, the pro-inflammatory chemokine Cxcl2 was significantly elevated in the small and large intestine of neonatal mice but elevated solely in the colon of DSS-treated adult animals.

An explanation for this finding may be the postnatal maturation of the small intestinal mucosa. Postnatally, a large variety of factors, including gut peristalsis, cellular maturation of the mucosal tissue, intestinal epithelial regeneration and the formation of tight junctions contribute to the establishment of the intestinal barrier [17–19]. Especially in preterm neonates, intestinal immune defense mechanisms are immature rendering the small intestine of these children more vulnerable to pathogens or harmful chemicals. Likewise, toll-like receptor-4 (TLR4) as part of a family of transmembrane innate immune receptor molecules and sensor for bacterial endotoxin has been shown to respond to induced inflammation in an age-dependent manner. Richardson et al. reported an increased enterocyte apoptosis by TLR4 activation through feeding and intraperitoneal injection of LPS in the ileum of newborn, but not adult mice [20]. In contrast, only marginal inflammation was observed in the colon in both age groups. TLR4 positive tissue macrophages were shown to directly control the synthesis of the neutrophil-attracting chemokine Cxcl2 following stimulation with LPS [21]. This is in line with our finding of an increased inflammatory response to DSS associated with an increased expression of Cxcl2 in the small bowel of neonatal mice and the large intestine of both adult and newborn animals.

Further, the size of the lesions after DSS treatment was significantly larger than in the LPS group. Whether the size of an intestinal lesion correlates with the severity of necrosis is unknown. However, the abundance of intraluminal erythrocytes as well as growth of intraluminal bacteria was significantly increased after DSS treatment, suggesting a stronger impact of DSS on intestinal inflammation in our model.

To further characterize our new model, we next investigated the humoral and cellular immune responses to DSS and LPS in detail. The humoral response was assessed by mRNA expression of Cxcl2 (MIP2) that acts as a critical chemokine for neutrophil recruitment and mediates intestinal inflammation and injury [22]. Cxcl2 is the murine analogue of human IL-8 which is highly elevated in human NEC, serving as a reliable marker for inflammation [19]. In the DSS group, Cxcl2 was significantly upregulated in the small and large intestinal tissue. In contrast, no increase was noted in animals that had received LPS or control animals. In line with these findings, Cxcl2 receptor (Ccxr2) knockout mice fail to develop colitis after DSS administration [23]. Thus, Cxcl2 appears to play a critical role in the pathogenesis of intestinal inflammation among other cytokines that are induced by DSS and LPS treatment in adult and neonatal animals [24–26]. This increase of Cxcl2 in colons of adult mice is in line with earlier studies [27].

To reveal the cellular inflammatory response to DSS, the composition of lamina propria resident leukocytes was analyzed by flow cytometry. NEC is characterized by an intestinal inflammation significantly mediated by the innate immune system. Thus, infiltration of neutrophils and macrophages/monocytes are important parameters in the pathogenesis of NEC [28]. DSS in adult mice presumably acts through breaching the intestinal barrier, which leads to an exposure of commensal bacteria to subepithelial immune cells, resulting in inflammation [29]. This process has been shown to be independent from lymphocyte actions, as mice lacking T cells and B cells still develop colitis after DSS treatment [29, 30].

Both, DSS and LPS treatment were associated with a significant decrease in the total number of viable leukocytes. A reduced number of viable leukocytes in a murine NEC model induced by LPS/hypoxia/hypothermia and formula feeding was previously shown by our group [11]. In fact, digested formula milk has been shown to have a toxic effect on intestinal cells [31], suggesting a potential contribution of formula constituents to the pathogenesis of NEC.

Analyzing leukocyte subpopulations, the amount of neutrophils in the *lamina propria* was significantly enhanced in the LPS model but no increase was noted after DSS treatment. However, the percentage of inflammatory (M1) monocytes/macrophages was significantly enhanced in both treatment groups in comparison to healthy controls and found to be particularly high after DSS exposure.

These results reflect a common inflammatory response, in which neutrophils infiltrate tissue first (as observed in the LPS group), but will be replaced by monocytes/macrophages in proceeding immune response (as shown in the DSS group) [32].

Increased numbers of inflammatory (M1) monocytes/macrophages in the *lamina propria* of newborn mice with LPS induced NEC have previously been reported [11, 33, 34]. In general, M1 monocytes/macrophages mediate the release of pro-inflammatory and pro-apoptotic cyto- and chemokines, which promotes the inflammatory response [22, 33]. Therefore, it has been speculated that infiltrating monocytes/ macrophages contribute to NEC through exaggerating the neonatal immune response by releasing pro-inflammatory cyto- and chemokines [35, 36].

We have previously shown that LPS/hypoxia/hypothermia over 72 h induces intestinal mucosal inflammation with NEC-like lesions [11]. Compared to the induction of NEC by LPS/hypoxia/hypothermia the inflammation induced by DSS administration developed after a shorter period and was even more pronounced.

To better understand the pathogenesis of the necrotic lesions, the viability of intestinal epithelial cells was examined. Unexpectedly, we could not observe a direct toxic effect of 3% DSS on the viability of primary small intestinal epithelial cells. This is in contrast to results from Araki et al., who observed cytotoxicity and an arrest of the cell cycle after direct exposure of 5% DSS, but of different molecular weight, to Caco-2 cells [37]. However, Caco-2 cells are mature, human epithelial colorectal adenocarcinoma cells, which are different to murine small intestinal cells. In addition this cell line does not represent the developmental stage of immature intestines found in neonatal mice, and was therefore not taken into consideration for investigating potential toxic effects on the small intestine [38].

Conversely, apoptosis was significantly predominant in caspase 3 staining after DSS treatment as compared to LPS treatment in neonatal mice. Thus, the observed apoptosis could be secondary to the formation of intestinal lesions rather than due to a primary toxic effect.

However, as changes of gut permeability due to dysfunctional tight junctions have been observed in adult mice treated with DSS a disturbance of the integrity of intestinal tight junctions might contribute to the development of NEC-like-lesions [39–41].

In this study, we are aware of some deficiencies regarding in depth investigations. These are difficulties obtaining required mRNA amounts for a larger primer panel, an analysis of blood parameters and a small sample size for the 36 h test series. In future studies, further analysis of different tight juction molecules and e-cadherin should be investigated precisely during the induction phase of NEC in the DSS model, which seems to be slightly before the 36 hour mark.

Despite these limitations we believe that the neonatal DSS model for the induction of NEC-like mucosal tissue lesions presented in this study maybe superior to the previously established LPS model in several aspects. First, our new model shows the histological features of NEC, accompanied by a significant humoral and cellular immune response. Second, no physical stressors like hypoxia and/or hypothermia are required, which exerts additional harm to the animals. Finally, the observed mucosal tissue changes occur after a relatively short time of DSS exposure. In conclusion our study demonstrates the potential of DSS to induce NEC-like histological lesions accompanied by a significant humoral and cellular immune response in the small and large intestine of neonatal mice.

## Supporting information

S1 Fig Macroscopic changes in the neonatal small intestine following exposure to LPS (a) or DSS (b and c). (a) LPS treatment did not cause macroscopic signs of necrosis but slight reddening. DSS treatment was associated with intraluminal blood collection and reddening of the small bowel (b) as well as necrotic parts (c). Whole organ preparation.
(TIF)

S2 Fig Intraluminal blood in the small bowel of neonatal mice after LPS and DSS treatment. (a) DSS treated animals showed (massive) intraluminal accumulation of blood (star), commonly accompanied by surrounding bacteria (2 stars). The evaluation (b) revealed a significant larger amount of intraluminal blood in DSS treated neonatal mice compared to LPS treatment. Magnification 200x, *** p < 0.001.
(TIF)

S3 Fig Lesion associated bacteria in the small bowel of neonatal mice after LPS and DSS treatment. Representative histological pictures illustrate bacterial overgrowth after DSS but not LPS treatment (a), which was significantly increased compared to LPS treatment (b). The most excessive bacterial overgrowth of a DSS treated neonatal mouse is shown in (c). Black arrows indicate areas of bacteria. Magnification 100x *** p < 0.001.
(TIF)
