# Supplementary figures and images for: Dextran sodium sulfate (DSS) induces necrotizing enterocolitis-like lesions in neonatal mice
Source: PLoS One. 2017 Aug 17;12(8):e0182732. doi: 10.1371/journal.pone.0182732 (PMC5560643; doi:10.1371/journal.pone.0182732)

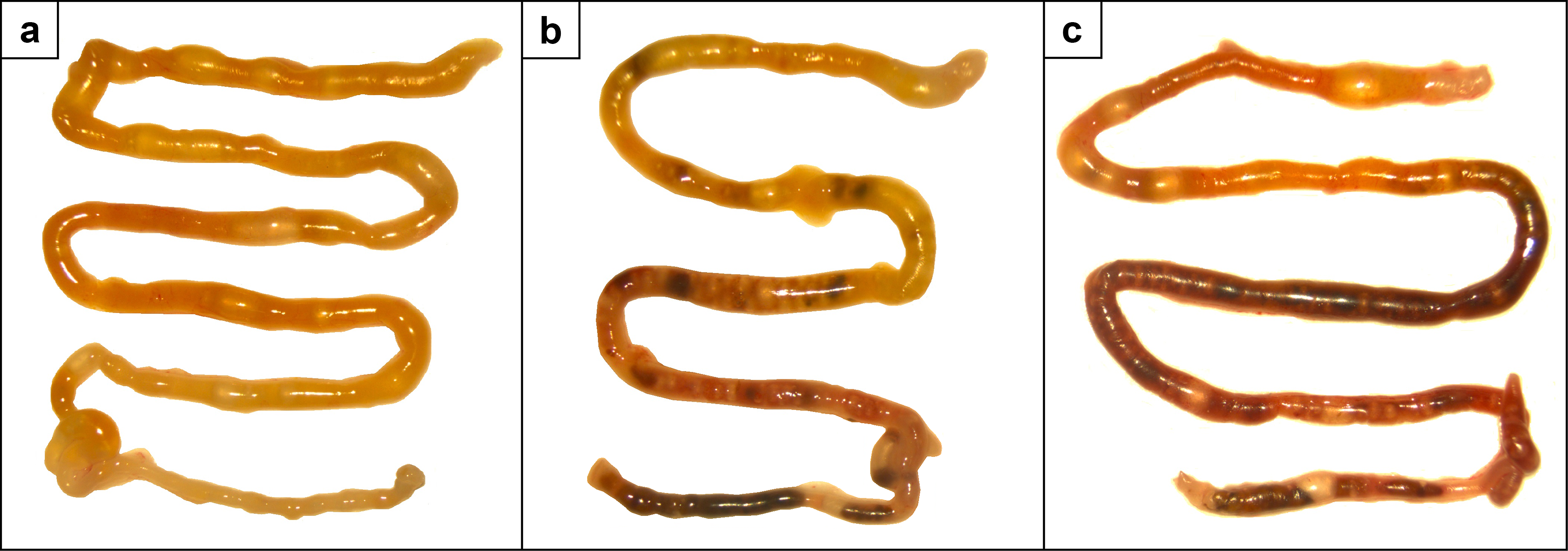

Supplement: S1 Fig — (a) LPS treatment did not cause macroscopic signs of necrosis but slight reddening. DSS treatment was associated with intraluminal blood collection and reddening of the small bowel (b) as well as necrotic parts (c). Whole organ preparation. (TIF) [file pone.0182732.s001.tif]

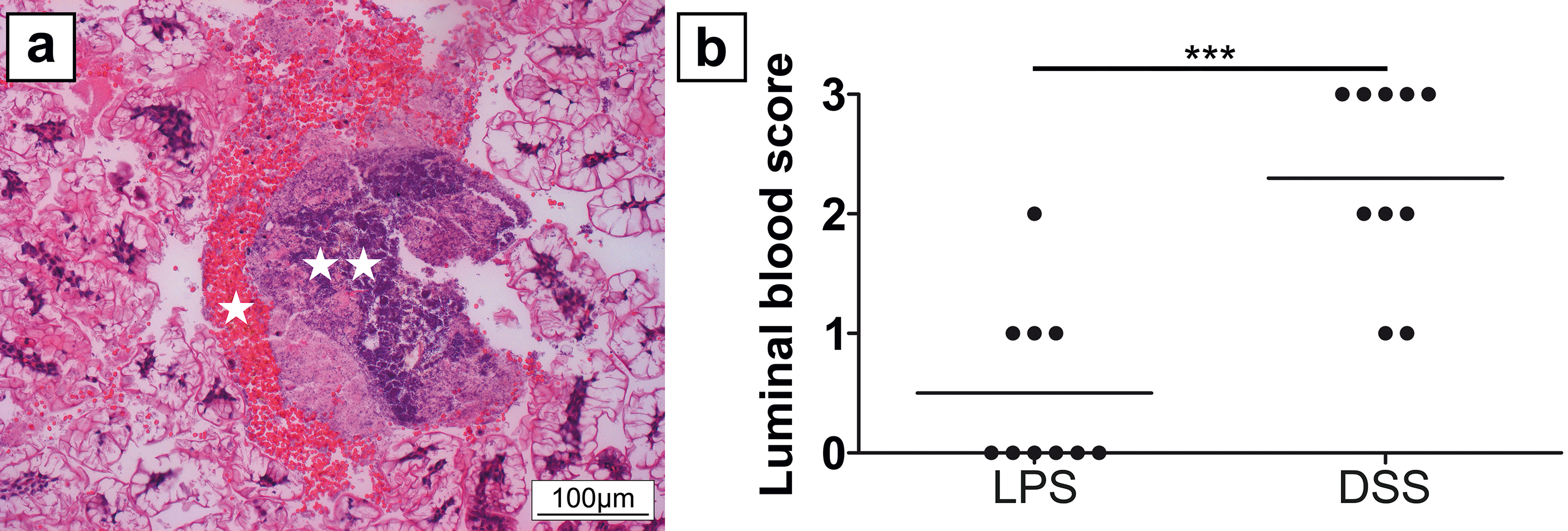

Supplement: S2 Fig — (a) DSS treated animals showed (massive) intraluminal accumulation of blood (star), commonly accompanied by surrounding bacteria (2 stars). The evaluation (b) revealed a significant larger amount of intraluminal blood in DSS treated neonatal mice compared to LPS treatment. Magnification 200x, *** p < 0.001. (TIF) [file pone.0182732.s002.tif]

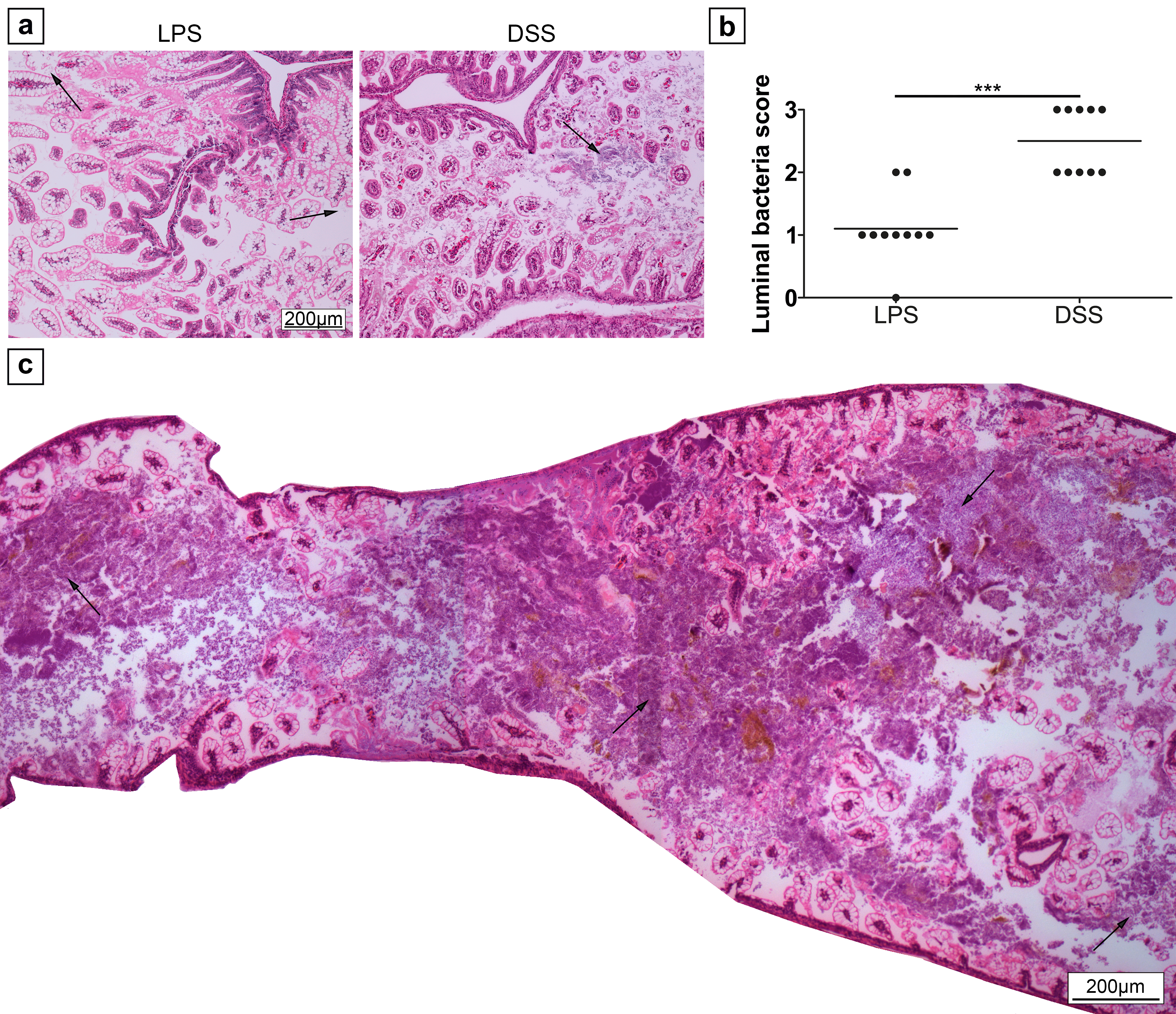

Supplement: S3 Fig — Representative histological pictures illustrate bacterial overgrowth after DSS but not LPS treatment (a), which was significantly increased compared to LPS treatment (b). The most excessive bacterial overgrowth of a DSS treated neonatal mouse is shown in (c). Black arrows indicate areas of bacteria. Magnification 100x *** p < 0.001. (TIF) [file pone.0182732.s003.tif]
